# Psychological Stress, Intestinal Barrier Dysfunctions, and Autoimmune Disorders: An Overview

**DOI:** 10.3389/fimmu.2020.01823

**Published:** 2020-08-25

**Authors:** Hanna Ilchmann-Diounou, Sandrine Menard

**Affiliations:** Neuro-Gastroenterology and Nutrition Team, Toxalim (Research Centre in Food Toxicology), Université de Toulouse, INRAE, ENVT, INP-Purpan, UPS, Toulouse, France

**Keywords:** intestinal permeability, psychological stress, type 1 diabetes, multiple sclerosis, systemic lupus erythematosus, microbiota, immune response

## Abstract

Autoimmune disorders (ADs) are multifactorial diseases involving, genetic, epigenetic, and environmental factors characterized by an inappropriate immune response toward self-antigens. In the past decades, there has been a continuous rise in the incidence of ADs, which cannot be explained by genetic factors alone. Influence of psychological stress on the development or the course of autoimmune disorders has been discussed for a long time. Indeed, based on epidemiological studies, stress has been suggested to precede AD occurrence and to exacerbate symptoms. Furthermore, compiling data showed that most of ADs are associated with gastrointestinal symptoms, that is, microbiota dysbiosis, intestinal hyperpermeability, and intestinal inflammation. Interestingly, social stress (acute or chronic, in adult or in neonate) is a well-described intestinal disrupting factor. Taken together, those observations question a potential role of stress-induced defect of the intestinal barrier in the onset and/or the course of ADs. In this review, we aim to present evidences supporting the hypothesis for a role of stress-induced intestinal barrier disruption in the onset and/or the course of ADs. We will mainly focus on autoimmune type 1 diabetes, multiple sclerosis and systemic lupus erythematosus, ADs for which we could find sufficient circumstantial data to support this hypothesis. We excluded gastrointestinal (GI) ADs like coeliac disease to privilege ADs not focused on intestinal disorders to avoid confounding factors. Indeed, GIADs are characterized by antibodies directed against intestinal barrier actors.

## Introduction

Autoimmune disorders (ADs) are multifactorial diseases involving, genetic, epigenetic, and environmental factors. In the past decades, there has been a continuous rise in the incidence of ADs, which cannot be explained by genetic factors alone. Changes in our lifestyle including diet, hygiene, exposure to social adversity, or pollutants have been suggested to be risk factors for ADs. ADs are associated with defect of the intestinal barrier; and besides nutrition, another environmental factor well described to impair the intestinal barrier is psychological stress. The aim of this review is to compile evidences highlighting a relationship between stress, intestinal barrier disruption, and occurrence of ADs. Even though no causative role of stress-induced intestinal barrier defect on AD onset has been demonstrated so far, the goal of this manuscript is to combine evidences on the basis of a review of the literature and offer a new field of research and perspectives on ADs. We will focus on three of the most studied ADs—autoimmune type 1 diabetes (T1D), systemic lupus erythematosus (SLE), and multiple sclerosis (MS)—for which we could find sufficient evidence supporting our hypothesis, that is, role of psychological stress and defect of intestinal barrier functions.

This review is based on epidemiological and preclinical studies. Numerous excellent and recent reviews treating either ADs and stress, or stress and the intestinal barrier will be quoted to support this hypothesis.

## Stress

Stress, firstly described in 1936 by Selye, is defined as a real (physical) or perceived (psychological) threat to homoeostasis, to which the organism has to react by an adaptive response (1).

Life time window, length, and frequency of exposure to stress play pivotal roles in their consequences on the individual pathophysiology. Indeed, acute and chronic stress exposure could occur in early life in a still maturating organism or at adulthood in mature organism. Traumatic experiences can lead to so-called post-traumatic stress disorder (PTSD), a condition in which the patient suffers from anxiety, depression, and flashbacks long after the traumatic experience (2). Persisting stress or inadequate response can lead to harmful maladaptive reactions depending on the kind of stress that will be discussed below. In this review, psychological stress is used as a general term that encompasses several psychological aspects (i.e., anxiety, depression, etc.).

The stress response is orchestrated by hypothalamic–pituitary–adrenal (HPA) axis and sympathetic nervous system (SNS). The neuroendocrine and autonomous responses are mediated by hormones such as epinephrine, norepinephrine, corticotropin-releasing hormone (CRH), adrenocorticotropic hormone (ACTH), glucocorticoids (cortisol in human and corticosterone in rodents) (3, 4). In the past, special attention has been paid to glucocorticoid in stress response and immune regulation. Endogenous glucocorticoids, part of the endocrine stress response, have ubiquitous functions in the development, metabolism, and inflammation. In general, glucocorticoids have been described to dampen immune response all along the inflammation process [for review, see (5)]: they attenuate signaling pathways of many pattern recognition receptors (6, 7), diminish leukocyte transmigration by reducing adhesion molecules (8), decrease the production of chemoattractants (9), program macrophages to anti-inflammatory M2c subtype (high expression of scavenger receptors and secretion of anti-inflammatory cytokines) (10), and decrease T cell response (11, 12), preferentially Th1 and Th17 by promoting Th2 and Treg (13, 14). Owing to their immunosuppressive effects, glucocorticoids have been used to treat various immune-related disorders like ADs.

The literature of the past 60 years has focused on immunosuppressive properties of glucocorticoids, but glucocorticoids can also enhance inflammation and immunity [for review, see (5)]. We will not go into the details of the diverging effects of glucocorticoid on immune response, but part of the explanation might reside in the diversity of glucocorticoid-receptors in different tissues, the presence or absence of 11βHSD, an enzyme inactivating cortisol, the time of glucocorticoid exposure (before or after tissue injury/inflammation) (15), and the dose (16). All those factors might explain that stressful events inducing glucocorticoids release could play a role in AD occurrence that can be treated by exogenous glucocorticoids. As an example, in humans, childhood maltreatment is associated with modified methylation of the glucocorticoid receptor gene NR3C1 in adults in brain and in leucocytes (17–19).

## Role of Stress in Autoimmune Disorders (20)

The onset of at least 50% of autoimmune disorders has been attributed to unknown trigger factors. Many retrospective studies observed that most of patients suffering from AD report uncommon emotional stress before disease onset (21). This is obviously a vicious cycle as AD causes stress in patients (22, 23). This review will focus on three of the most studied ADs that will provide sufficient evidence to support the hypothesis of a role of stress-induced intestinal barrier defect on AD onset, that is, T1D, SLE, and MS. T1D is characterized by a defect of insulin production by pancreas owing to an autoimmune response against host pancreatic β-cells (24). SLE is an AD characterized by severe and persistent inflammation that leads to tissue damage in multiple organs (25, 26). MS is a chronic disease affecting the central nervous system and characterized by a defect of the blood–brain barrier and demyelination of the neurons of the central nervous system due to infiltration of auto-reactive T cells (27, 28). The most widely used preclinical MS model is experimental autoimmune encephalomyelitis (EAE).

A potential association between stressful events and T1D has been highlighted already a long time ago when Thomas Willis links, in 17th century, T1D onset to prolonged sorrow (29). Early life stress seems to be of particular risk for T1D development (30, 31). This is in accordance with literature highlighting neonatal maturation of pancreas as critical and vulnerable to stressors (32). Stress in adult has been described to increase incidence of SLE (33) and is able to exacerbate SLE symptoms (physical pain, sleep disturbances, and unemployment) (34). Around 70% of MS patients reported unusual amount of stress before the onset of the disease (35, 36).

Those epidemiological studies suggest that stress could be involved in both triggering and exacerbating ADs. Whether it is dependent on the kind of stress or ADs involved is unknown, and it would be interesting to conduct both retrospective epidemiological studies and preclinical studies to better document the role of stress in ADs. However, some interventional studies suggest that stress management could benefit to AD patients. Indeed, escitalopram (antidepressant) decreases the risk of MS relapsing in women (37). Diazepam (tranquilizer) decreases EAE incidence and histological signs associated with this disease in a mouse model (38). A meta-analysis of 21 trials showed that in the 10 studies of children and adolescents with supportive or counseling therapy, cognitive behavioral therapy and family system therapy reduced glycosylated hemoglobin and as such improved diabetes control (39). Interestingly, in the 11 studies in adults, no beneficial effect of stress management could be observed on T1D (39).

## Consequences of Stress on Intestinal Barrier and Systemic Immune Response

Stress can affect various physiological processes. Already Selye observed and others confirmed that the gastrointestinal tract and the immune system are particularly responsive to stress no matter the origin of the stress (1).

### Actors of Intestinal Barrier and Function

Intestinal epithelium is the mammalian organism's biggest surface in contact with the environment. Therefore, intestinal barrier functions are highly diverse and well developed. The intestinal barrier has to fulfill conflicting functions. Indeed, the intestinal barrier allows the transport of nutrient but at the same time filters and defends the organism from harmful luminal content (pathogens, toxins, etc.). Among the main actors of the intestinal barrier we can quote, intestinal microbiota, intestinal epithelium, and immune response (innate and adaptive). All those actors are in close relationship and regulate one another [for review, see (40)]. Intestinal microbiota not only participates in the protection against pathogens colonization but also contributes to maturation of intestinal epithelium and immune system and provides various nutritional compounds (41). The intestinal epithelium is formed by distinct cell types distributed along the crypt–villus axis. Although they all derive from a common stem cell progenitor located in the crypts, their morphology and roles differ [for review, see (42)]. The intestinal epithelium is renewed every 5 days, and this constant renewing confers high plasticity and protection to the intestinal barrier because defective cells are removed rapidly (43). Intestinal permeability is the ability of intestinal epithelium to allow the selective entrance of luminal antigens into the organism (44). Another actor of the intestinal barrier is the intestinal immune system. Gut-associated lymphoid tissue (GALT) represents the inductive site for B and T cells of mucosal intestinal barrier and includes the Peyer patches (PPs), the appendix, and isolated lymphoid follicles (ILFs). The humoral response in the intestines can be divided into four stages: predominant IgA induction in mucosal B cells, recirculation of IgA plasma blasts and homing into the intestinal mucosa, terminal B cell differentiation to plasma cells with local IgA production, and export of IgA through the intestinal epithelial layer [for review, see (45)]. Most intestinal T cells mature in peripheral lymphoid organs where they acquire the expression of intestinal homing receptors to migrate to the effector site of the intestines, that is, the mucosal epithelia and the *lamina propria*. Intestinal lymphocytes are continuously exposed to food and microbial antigens. These lymphocytes help to maintain the integrity of the intestinal barrier and immune homeostasis. Owing to their close location to luminal antigens, they have dual functions: regulatory functions (i.e., maintaining tolerance toward food antigens and commensal microbiota) and effector functions (i.e., prevention of pathogenic invasion) [for review, see (46)]. Innate lymphoid cells (ILCs) are lymphocytes that do not express the type of diversified antigen receptors expressed on T cells and B cells. ILCs are largely tissue-resident cells participating in tissue homeostasis [for review, see (47)]. A defective intestinal barrier will lead to inappropriate intestinal but also systemic immune response leading to gastrointestinal disorders and to extra-intestinal diseases like autoimmune diseases (48–50).

Intestinal barrier homeostasis is highly regulated, and a defect in microbiota composition could lead to intestinal hyperpermeability and intestinal inflammation. Intestinal inflammation not only will contribute to intestinal hyperpermeability (51) but also will favor microbiota colonization by pathobionts (52). Microbiota, intestinal permeability, and immune response mutually regulate one another making it difficult to define their respective role as cause or consequence in complex established pathologies.

### Psychological Stress Impairs Intestinal Barrier

**Figure 1:**
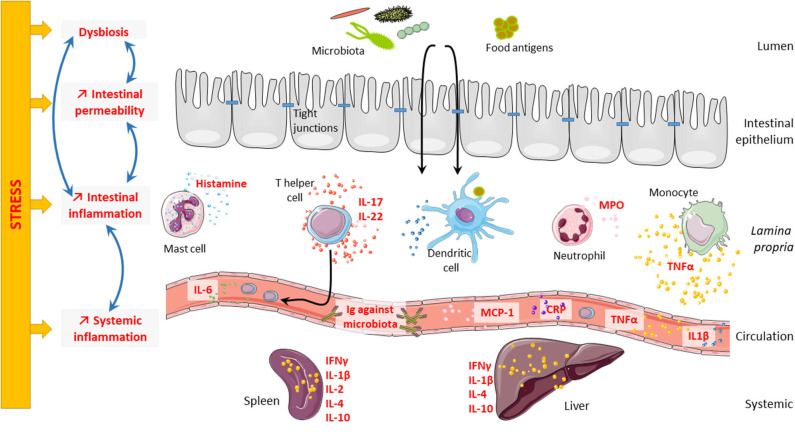
Consequences of stress on intestinal barrier and systemic inflammation. Psychological stress can impair intestinal barrier at different levels. Indeed, stress can lead to microbiota dysbiosis, intestinal hyperpermeability, and intestinal inflammation. Interestingly, all these elements are highly connected and regulate one another. Microbiota dysbiosis can trigger intestinal hyperpermeability and intestinal inflammation; and in contrast, both intestinal hyperpermeability and intestinal inflammation can induce microbiota dysbiosis. Finally, stress can also induce systemic inflammation that might be related to intestinal inflammation.

Stress plays a role in the course of gastrointestinal disorders like irritable bowel syndrome (IBS) (53–55) and inflammatory bowel disease (IBD) (56). IBS is a very interesting model to study the consequences of stress on the intestinal barrier. Indeed, the occurrence of stressful events is considered as a contributing factor triggering and/or maintaining IBS (57, 58), suggesting that dysfunctional interactions in the brain–gut axis contribute to the pathophysiology of the disease (59) and as such justifying its new classification as a disorder of the brain–gut interaction (60). In this review, we will focus on the consequences of psychological stress on the intestinal barrier and its consequences on systemic immune response (Figure 1).

#### Microbiota Dysbiosis

Stress is modifying microbiota in animal and human. Neonatal maternal separation induces microbiota dysbiosis in mice at different ages (61, 62). Limited nesting stress alters microbiota in rat pups (63). In adults, chronic water avoidance stress increases susceptibility to indomethacin-induced hyperpermeability in mice, and the effect is transferable *via* fecal microbiota transfer (64). Germ-free (GF) mice have exaggerated HPA stress response after restraint stress (65), showing the role of microbiota in the regulation of stress response. Mice exposed to social disruption stress have increased circulating IL-6 and MCP-1 levels; these effects were totally abolished by antibiotic treatment showing the importance of microbiota in the induction of stress effects (66).

In humans, decreased total abundance of Actinobacteria, Lentisphaerae, and Verrucomicrobia is associated with PTSD in South African individuals (67). Microbiota dysbiosis has been described in IBS patients [for review, see (68)].

#### Stress Is Associated With Intestinal Hyperpermeability

In preclinical models and epidemiological studies, stress has been associated with an increase of intestinal permeability. Chronic water avoidance stress increases intestinal permeability and decreases tight junction protein expression in colon of adult rat (69) and overall intestinal permeability in mice (70). Chronic neonatal maternal separation, a model of early life stress, also increases intestinal permeability in rat (63, 71, 72) and mice (73). Maternal separation applied just for one time (acute stress) increases intestinal permeability in rats (74). Combination of different stressors [subacute (isolation, limited movement) and chronic crowding stress] also decreases tight junction mRNA expression in rats (75). In a mouse model of social disruption, a social stressor, bacterial RNA (*Lactobacillus* spp.), is increased in spleen, which indicates bacterial translocation (76).

In human, acute psychological stress like public speaking has also been shown to induce intestinal hyperpermeability (77). Intestinal hyperpermeability has also been described in IBS patients (78). The stress hormones cortisol (human) and corticosterone (mice) have been shown to mediate stress increased intestinal hyperpermeability as administration of the GR agonist dexamethasone mimics the intestinal hyperpermeability (69, 74).

#### Stress Exacerbates Intestinal and Systemic Inflammation

Chronic neonatal maternal separation in rats increases cytokine expression, myeloperoxidase activity, and mast cell numbers in colonic tissue and exacerbate TNBS-induced colitis (71). Neonatal maternal separation in mice increases TNFα expression by intestinal tissue in young adult (61) and lipopolysaccharide (LPS)-stimulated TNFα secretion of isolated *lamina propria* immune cells in aging (62). Acute restraint stress augments histamine release by mast cells (79). Acute acoustic stress increases intestinal IL-17 and IL-22 expression in mice (80).

In human, stress aggravates IBD symptoms including higher release of pro-inflammatory effectors (56). In IBS, an increased state of activation of immune cells has been described even though this observation is under debate (81).

Not only the intestinal immune system is influenced by psychological stress, but there is also evidence for modified systemic immune response without direct proof that inflammatory immune cells were activated in the gastrointestinal tract. Neonatal maternal-deprived rats have increased cytokine expression in liver and spleen (71). Humoral immune response against microbiota is increased in neonatal maternal-deprived mice (62, 73). Social disruption stress in mice increases bacterial translocation and induces circulating IL-6 and MCP-1 (66, 76).

Stress is associated with an increase in pro-inflammatory response as described in PTSD patients (82). A meta-analysis of several studies showed that IL-6, TNFα, and IL-1β secretion are increased in response to acute stress in human (83). Childhood victimization is associated with elevated C-reactive protein (CRP) levels in young adult (84, 85).

## Defect of Intestinal Barrier in Autoimmune Disorders (86)

**Figure 2:**
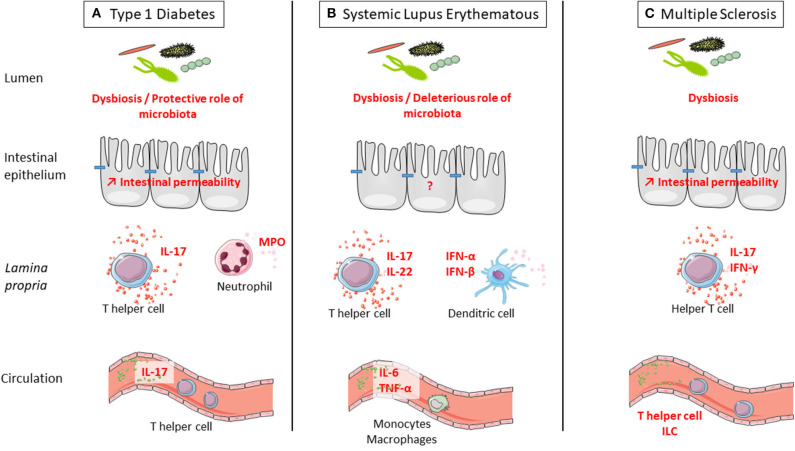
Defect of intestinal barrier and systemic immune response is observed in three examples of autoimmune disorders (ADs): type 1 diabetes (T1D), systemic lupus erythematosus (SLE), and multiple sclerosis (MS). **(A)** T1D is associated with microbiota dysbiosis, intestinal hyperpermeability, increased IL-17 secretion in the intestine and at systemic level, and increased myeloperoxidase (MPO) in the intestine. Interestingly, colonization by a complex microbiota is protective from type 1 diabetes. **(B)** SLE is associated with microbiota dysbiosis and increased secretion at the intestinal level of IL-17 and IL-22 by T cells and IFN-α and IFN-β by dendritic cells. At the systemic level, there is a higher secretion of IL-6 and TNF-α by monocytes and macrophages in SLE. Interestingly, colonization by a complex microbiota is deleterious for SLE onset. **(C)** MS is associated with microbiota dysbiosis, intestinal hyperpermeability, and increased secretion at the intestinal level of IL-17 and IFN-γ by T cells. At the systemic level, the number of innate lymphoid cell (ILC) population was observed in MS. Interestingly, colonization by a complex microbiota is deleterious for MS onset.

We provided evidence that stress might play a role in onset or course of ADs, and we reviewed the well-documented deleterious role of stress in intestinal barrier functions. We will now summarize the data regarding the defect of the intestinal barrier in ADs. Indeed, the observed defect of the intestinal barrier in ADs is an interesting lead that largely contributes to the rise of the hypothesis, suggesting a contribution of stress-induced intestinal barrier defect in ADs (Figure 2).

### Microbiota in Autoimmune Disorders

Microbiota is known to contribute to intestinal mucosal permeability and induction of innate defenses and as such represent a risk factor for ADs (87, 88). A growing body of evidence suggests that intestinal microbiota can affect the incidence and/or severity of immune-mediated extra-intestinal diseases (89). Aberrant microbiota has been described in patients suffering from T1D (90), SLE (88), and MS (91). Knowledge regarding microbiota dysbiosis in AD patients and animal models will be summarized here, and interventional studies, which help to understand the role of microbiota in those diseases, will be discussed at the end of the paragraph.

Increased microbial diversity and low level of butyrate have been observed in feces of pediatric T1D patients (92, 93). In the BABYDIET cohort, early development of islet auto-antibodies is associated with alteration in the composition of mucin-degrading bacteria, that is, increase of Bacteroides and decrease of *Akkermansia* (94). A reduction of microbial diversity is more pronounced before the time of diabetes onset (95). Fecal transplantation of NOD diabetic microbiota in NOD-resistant mice induced insulitis, suggesting a diabetogenic gut microbial community (96, 97). Antibiotic treatment accelerates disease development (98, 99), suggesting a protective role of microbiota colonization in T1D.

Microbiota dysbiosis has been observed in relapsing–remitting MS patient compared with healthy control (100–103) with no consensus on the involvement of a particular bacterial species. In contrast, another study comparing 16S RNA profiles of feces from MS and healthy patients has not shown any differences (101). Demyelination initiates after colonization with feces of specific pathogen-free mice (104). Microbiota depletion by non-absorbable antibiotics delays the development of EAE by reducing the number of mesenteric Th17 cells (105). GF mice present attenuated symptoms in both spontaneous and induced EAE models (104, 106), suggesting a deleterious role of microbiota colonization in MS. Furthermore, microbiota shapes and predicts the course (chronic-progressive or relapsing–remitting) of EAE in a mouse model (107).

Only a few studies on human SLE microbiome in small cohorts report microbial dysbiosis (108–110), but they are confirmed by preclinical studies in mouse models (110). A study performed in a larger and diversified cohort of SLE patient showed that the severity of disease is associated with more severe microbiota dysbiosis (111).

#### Interventional Studies: What Do They Tell Us?

Here, we will focus on direct supplementation by living bacteria like probiotic and fecal microbiota transplantation (FMT) treatment but not on indirect interventions like prebiotics or nutritional compounds produced by bacteria, as short chain fatty acids, for example, which may involve indirect effects. Once ADs are diagnosed, the production of antibody against self-antigen will remain and will still damage tissues, but this process could be delayed or reduced. Probiotics are living microorganisms that confer a health benefit to the host (112). Probiotics are known to have beneficial effects on the intestinal barrier (113, 114) and anti-inflammatory properties (115–119) and as such represent an interesting tool to delay, reduce, or even prevent ADs.

Animal studies suggest beneficial effects of probiotics supplementation on EAE *via* a stimulation of IL-10 production (106, 120–124). Clinical studies showed that a mixture of probiotics improves expanded disability status score and decreased inflammatory markers (125). Regarding T1D, probiotic treatment delays the onset of T1D in an experimental rat model and improves the intestinal barrier (126). Probiotics also protect NOD mice from T1D by reducing intestinal inflammation (127). In humans, it has been demonstrated in the TEDDY (The Environmental Determinants of Diabetes in the Young) cohort that early probiotic supplementation is associated with a decreased risk of islet autoimmunity as compared with late or missing supplementation (128). In animal models for lupus nephritis, probiotic administration lowers inflammatory response in the kidney and intestines in female and castrated males but not in non-castrated males (129).

FMT with microbiota from different diabetes resistant mouse strains delays the onset of T1D in NOD diabetes-prone mice (97). Few studies investigate to role of FMT on AD symptoms, and most of the time the recommendation for FMT treatment was to target associated gastrointestinal troubles. Neurological symptoms are improved and MS progression is paused in three MS patients who underwent FMT treatment for chronic constipation (130). Unfortunately, no data are available on intestinal barrier functions of probiotics and FMT treatments in parallel to beneficial effects on ADs.

#### Mimicking

Antigens from infectious agents and myelin proteins can share structural similarities called molecular mimicry. This molecular mimicry can be responsible for activation of naïve autoreactive T cells recognizing peptides from infectious agents but also from self-antigens myelin proteins. Cross-reactivity could occur when important motifs are conserved and overall structures of TCR–peptide–MHC interaction are similar, suggesting that cross-reactivity may happen frequently (131). Myelin basic protein, the immunodominant autoantigen of MS, cross react with Epstein–Barr virus (EBV), influenza A virus, herpes simplex virus, human papilloma virus (132), or human herpesvirus-6 (133). Regarding EBV, MS patients seem to have increased antibody titers against certain antigens of the virus than have control subjects even before the onset of MS (134). Despite cross-reactivity, infectious agents can impair self-antigen tolerance by indirect activation (135). It has been showed that an integrase expressed by intestinal Bacteroides encodes a low-avidity mimotope of the pancreatic β-cell autoantigens and as such might participate to T1D onset. Colonization of GF mice with Bacteroides promotes the recruitment of diabetogenic CD8^+^ T cells to the gut (136).

### Intestinal Hyperpermeability in Autoimmune Disorders

In ADs, intestinal hyperpermeability has been described, resulting in an increased entry of luminal antigens derived from food and/or intestinal microbiota or pathogens. The associated inflammation has been suggested to participate in AD onset and/or exacerbation.

Even though it is still unclear whether intestinal hyperpermeability is a trigger or a consequence of T1D progression (93, 137, 138), epidemiological and preclinical studies demonstrated that intestinal hyperpermeability occurs before disease onset (139, 140). Reversion of intestinal hyperpermeability by treatment with a zonulin 1 (intestinal homolog of a *Vibrio cholerae* enterotoxin, which reversibly increases intestinal permeability) inhibitor ameliorates T1D manifestation in rat model (141). Microbial translocation in pancreatic lymph nodes activates NOD2, and IL-17 production in pancreatic lymph nodes and pancreas which contributes to T1D development (142).

Intestinal hyperpermeability precedes EAE onset and increases while disease progresses (143). In this model, increased intestinal permeability is associated with the increase of crypt depth and mucosa thickness in jejunum and ileum, as well as with an overexpression of zonulin 1 (143) as observed for T1D (141, 144).

Intestinal barrier defect and subsequent exposure to microbial products play an important role in the pathology of SLE (145, 146). sCD14, lysozyme, and CXCL16 are markers of antimicrobial response found increased in SLE subject attesting to a defect of the intestinal barrier (147).

### Intestinal Inflammation

T1D is associated with increased intestinal myeloperoxidase activity and goblet cell (producing mucus) density, supporting the idea that early intestinal inflammation might lead to intestinal hyperpermeability (148, 149). Many studies suggest that the increased number of Th17 cells is involved in the pathogenesis of autoimmune diabetes. Higher numbers of IL-17 secreting cells are detected in recent-onset T1D-promoting inflammatory response to β-cells (150, 151). Th17 is increased in the peripheral blood of children with T1D (151, 152). *In vitro* IL-17 potentiates inflammatory and proapoptotic responses on human islets cells (151). Anti-IL-17 treatment reduces islet T cell infiltrates and GAD65 autoantibodies in NOD mice (153). Neutrophil extracellular traps (NETs) might contribute to the generation of ADs by exposing autoantigen (154). The role of NET has been studied particularly in T1D. Indeed, degradation of NETs in the gut prevents immune infiltration of pancreatic islet preserving β-cell mass and systemic inflammation (155).

In a mouse model of SLE developing severe nephritis, α4β7 expressing T cells is increased in PPs and pro-inflammatory cytokines (IL-17, IL-22, IFNα, and β) are much more expressed in distal ileum (156). Furthermore, intestinal monocytes/macrophages of SLE patients have an altered expression of type 1 interferon-stimulated genes, HLA-DR, and Fcγ receptors (157, 158). Monocytes isolated from plasma of SLE patients release higher pro-inflammatory cytokines in response to LPS than do healthy patients (159). More generally, higher production of pro-inflammatory cytokines by monocytes/macrophages has been described in SLE patients [for review, see (160)].

In MS, elevated Th1 and Th17 pro-inflammatory responses are observed in *lamina propria*, PPs, and mesenteric lymph nodes (143). GF EAE animals produce lower levels of IFNγ and IL-17 in the intestines associated with a higher number of Treg cells (104). Monocolonization of GF animals with segmented filamentous bacteria, IL-17 inducer in gut (161, 162), induces EAE and shows that microbiota can affect neurologic inflammation by recirculation of Th17 to the brain, causing inflammation (106). Autoreactive T cells from gut could migrate in different organs depending on pathologies, to brain in the case of MS, to liver in the case of autoimmune cholestatic liver disease (163, 164), or to the kidney in the case of SLE (165). Interestingly, not only T cells seem to be involved in MS but also circulating ILC. Indeed, a higher number of ILC have been observed in MS patients (166).

## Conclusion

As a conclusion, compiling evidences highlight the importance of both intestinal barrier defect and stress in ADs. Stress is well known to have long-lasting deleterious consequences on the intestinal barrier. A transversal research on ADs, stress, and intestinal barrier function would be of great interest and would bring new understanding in the pathophysiology of ADs. Identifying stress-induced intestinal barrier dysfunction as an actor of ADs could bring new possibilities for therapeutic targets and especially preventing strategies toward the spreading epidemic of ADs. Therapeutic strategies suggest that probiotics and FMT treatment might improve AD symptom, but preventive strategies in an at-risk population still need to be explored. In this review, we did not mention autoimmune thyroid diseases (AITDs) that are the most frequent ADs (167). It is difficult to study AITDs by themselves, as they are often observed together with other ADs, which are named polyautoimmunity (168). Then, even though there are sufficient data supporting the role of stress in AITD onset (20), evidences for a defect of intestinal barrier functions in AITD are sparse, and only two studies are available regarding microbiota dysbiosis (169, 170). For those reasons, we did not use AITD to illustrate the hypothesis of this review, supporting a role of stress-induced intestinal barrier disruption in the onset and/or the course of ADs. However, we wanted to mention the case of AITD as data on intestinal barrier function would be of great interest in the future.

## Author Contributions

HI-D and SM reviewed the literature, wrote and corrected the manuscript, and drew the figures. All authors contributed to the article and approved the submitted version.

## Conflict of Interest

The authors declare that the research was conducted in the absence of any commercial or financial relationships that could be construed as a potential conflict of interest.
